# Associations between El Niño-Southern Oscillation (ENSO) and tuberculosis incidence in China: a nationwide study

**DOI:** 10.1016/j.onehlt.2026.101587

**Published:** 2026-09-16

**Authors:** Xiaopeng Qin, Yizhang Xia, Jiang Xue, Xudong Zhou, Wenjie Tao, Yukang Wu, Hongchang Zhang, Ying Chen, Zhenyu Lin, Lanwei Nong, Kechang He, Zhendong Qin, Tiejun Zhou, Zhouhua Xie, Jie Ma

**Affiliations:** a Subcenter of the National Clinical Research Center for Infectious Diseases, HIV/AIDS Clinical Treatment Center of Guangxi (Nanning), The Fourth People's Hospital of Nanning, Nanning, Guangxi 530023, PR China; b State Key Laboratory for Infectious Disease Prevention and Control-Zigong Research Base for Emerging Infectious Diseases, Zigong Center for Disease Control and Prevention, Zigong, Sichuan 643000, PR China; c Guangxi Medical University, Nanning, Guangxi 530021, PR China; d Institute of Hydraulic and Ocean Engineering, Ningbo University, Ningbo, Zhejiang 315021, PR China; e School of Management, Xi'an Jiaotong University, Xi'an, Shaanxi 710049, PR China; f The Affiliated Wuxi Center for Disease Control and Prevention of Nanjing Medical University, Wuxi Center for Disease Control and Prevention, Wuxi, Jiangsu 214023, PR China; g College of Life Sciences, University of Chinese Academy of Sciences, Beijing 100049, PR China

**Keywords:** El Niño-Southern Oscillation (ENSO), Tuberculosis, Climate change, Distributed Lag Non-Linear Models (DLNMs), One Health

## Abstract

**Background:**

Climate variability may influence tuberculosis (TB) burden, but associations between the El Niño-Southern Oscillation (ENSO) and TB incidence remain poorly characterized across China.

**Objective:**

To quantify associations of ENSO and meteorological factors with TB notification incidence and assess regional and age-specific heterogeneity.

**Methods:**

Monthly TB surveillance data from 31 provincial-level administrative divisions in mainland China during 2005–2020 were analyzed using quasi-Poisson regression with distributed lag nonlinear models. Meteorological variables were examined alongside ENSO, with adjustment for relevant environmental and socioeconomic covariates. Subgroup analyses were conducted by geographic region and age group.

**Results:**

ENSO-related associations emerged at approximately 9 months, peaked at 10 months, and persisted for about 3 months. At lag 9 months, strong El Niño and strong La Niña conditions were associated with higher TB notification incidence than neutral conditions, with relative risks (RRs) of 1.048 (95% CI, 1.007–1.091) and 1.058 (95% CI, 1.032–1.085), respectively. Temperature showed a U-shaped association with TB notification incidence. Relative humidity of 67–87% was associated with higher TB notification incidence, peaking at 81% (RR, 1.101; 95% CI, 1.063–1.140). Regional and age-stratified analyses indicated heterogeneous exposure-response patterns.

**Conclusions:**

ENSO and local meteorological factors were associated with TB notification incidence through nonlinear and delayed exposure-response patterns. Climate forecasts may provide supplementary information for climate-sensitive TB surveillance within the environmental dimension of One Health.

## Highlights

• This study examines associations between ENSO and TB notification incidence in China.
• ENSO-related associations emerge after approximately 9 months and peak at 10 months.
• Temperature shows a U-shaped association with TB notification incidence.
• Relative humidity of 67% to 87% is associated with higher TB notification incidence.
• Regional and age-stratified analyses show heterogeneous exposure-response patterns.

## Graphical abstract

The figure was generated with support from the FIGDRAW platform.

**Figure:**
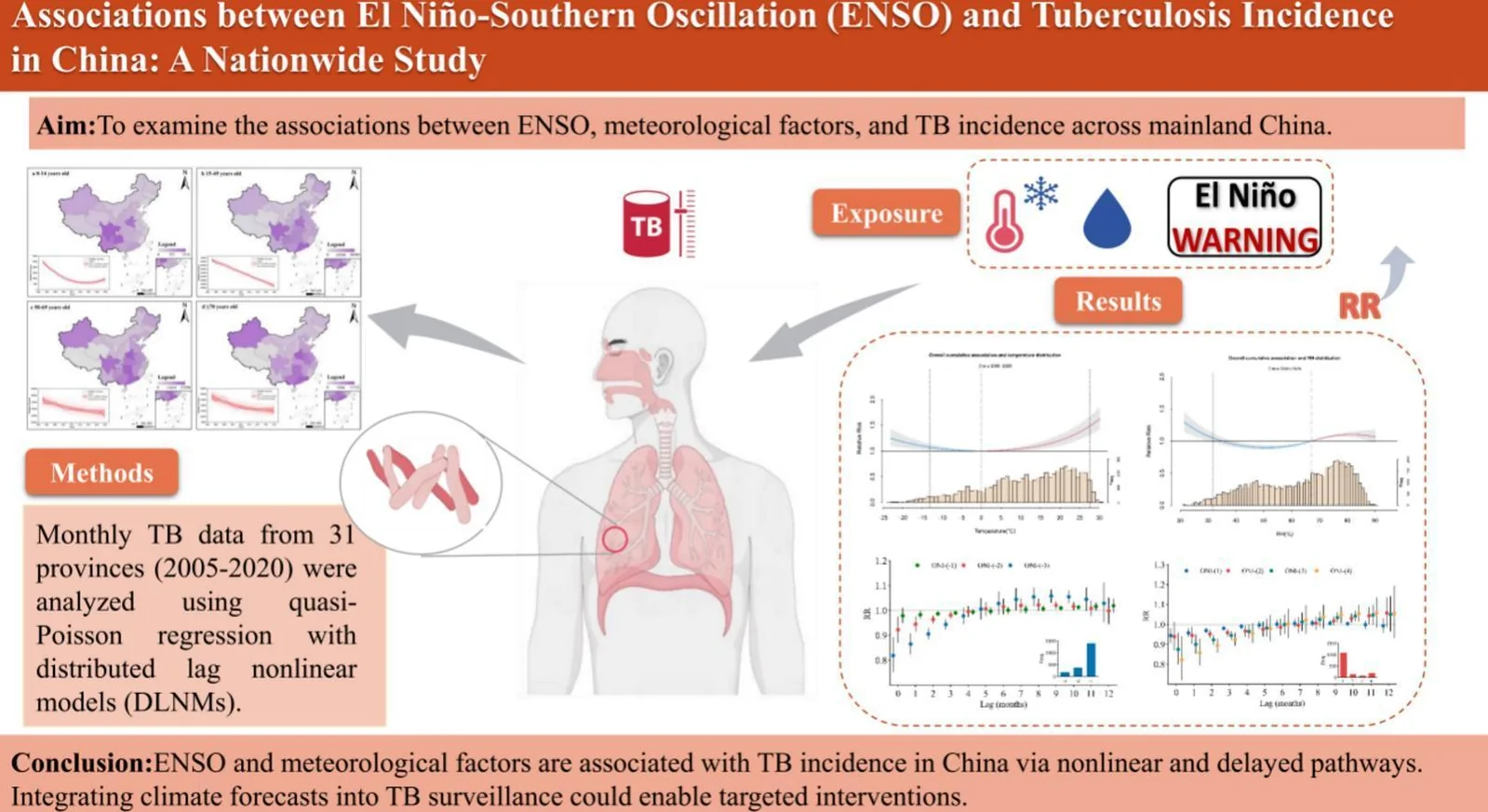

## List of abbreviations

**Table:** 

El Niño-Southern Oscillation	ENSO
Tuberculosis	TB
Distributed Lag Non-Linear Models	DLNMs
Oceanic Niño Index	ONI
Sea Surface Temperature	SST
Chinese Center for Disease Control and Prevention	CDC
National Oceanic and Atmospheric Administration	NOAA
European Centre for Medium-Range Weather Forecasts	ECMWF
Relative Humidity	RH
Fine Particulate Matter	PM_2.5_
Per Capita Gross Domestic Product	PGDP
Relative Risk	RR
Confidence Interval	CI
and *Mycobacterium tuberculosis*	MTB

## 1 Introduction

Climate change is increasingly recognized as a determinant of infectious disease distribution and burden [1]. Tuberculosis (TB) remains a leading cause of death from a single infectious agent, with marked geographical heterogeneity across China [2], [3]. Meteorological conditions can influence TB transmission through effects on pathogen survival, host susceptibility, and human behavior, often in nonlinear and time-lagged ways [4]. Given China's substantial climatic and socioeconomic heterogeneity, the setting is well-suited to examine how climate-sensitive TB risk varies by region [5], [6], [7].

Despite growing evidence on meteorological influences, the role of large-scale climate variability remains insufficiently characterized. Short temporal windows and linear specifications may miss nonlinear and delayed effects and obscure spatial heterogeneity [8], [9].

Although evidence is accumulating regarding climate-sensitive infectious diseases, research that explicitly examines the effects of the El Niño Southern Oscillation (ENSO) on TB remains limited. Most existing studies on TB have primarily focused on short-term associations with local meteorological factors, often within limited temporal windows, and have rarely considered large-scale climate variability that captures persistent macroclimatic conditions and delayed population-level responses. Foundational reviews established that El Niño events can influence population health across multiple regions, while more recent work shows that ENSO can shape respiratory epidemics when evaluated with local meteorology and can increase nutritional vulnerability linked to TB susceptibility [10], [11], [12], [13]. These observations suggest that ENSO may be relevant to TB even without a direct one-to-one correspondence with a specific meteorological parameter.

Growing evidence indicates that ENSO may influence infectious disease risk by reshaping the broader environmental and vulnerability context in which transmission occurs. Atmospheric boundary layer structure and stagnation conditions can affect near-surface aerosol accumulation and local exposure environments [14], [15]. These processes provide a plausible environmental pathway through which large-scale climate variability may modify respiratory exposure conditions, although this mechanism was not directly evaluated in the present study. Furthermore, climatic disruptions associated with ENSO phases can restructure exposure opportunities through population displacement and crowding [16], [17]. In parallel, ENSO-driven nutritional stress can weaken host immunity [18], [19]. These pathways provide a conceptual rationale for integrating ENSO with local meteorological indicators when evaluating climatic determinants of TB in a geographically diverse setting such as China.

Beyond these pathways, a broader body of climate epidemiological evidence supports the expectation that ENSO can modulate TB risk through interacting environmental, physiological, and social mechanisms. At the environmental level, ENSO affects atmospheric stability and humidity persistence, which condition airborne transmission potential [20], [21]. At the physiological level, ENSO-associated nutritional stress impairs immune capacity and increases susceptibility to infection [12], [22]. At the social level, ENSO-related hydrological shocks increase crowding and displacement, reshaping human contact patterns in high-risk contexts [16], [23]. Taken together, these mechanisms justify examining ENSO as a macroclimatic determinant of population susceptibility that interacts with local meteorological exposure to shape TB risk.

By moving beyond single-meteorological exposures, this study evaluates ENSO as a macroclimatic driver of TB risk, characterizes its nonlinear and lagged associations using distributed lag nonlinear models, and identifies regional and age-specific vulnerabilities that are not readily captured by conventional weather-based analyses. In this study, we used monthly TB surveillance data from 31 provinces in mainland China between 2005 and 2020, alongside high-resolution meteorological data and ENSO indices, to examine the associations between ENSO, key meteorological factors, and TB incidence. We applied distributed lag nonlinear models within a quasi-Poisson regression framework to assess nonlinear exposure lag response relationships. Subgroup analyses were conducted by geographic region and age group to identify potential modifiers of these associations.

This study extends existing meteorology-focused evidence by integrating a macroclimatic teleconnection with local meteorological conditions, using nationwide surveillance data to characterize nonlinear and delayed associations. It quantifies exposure thresholds and identifies nationwide and region-specific lag patterns, and demonstrates elevated TB risk during both El Niño and La Niña phases. This approach addresses limitations common to short study windows, linear specifications, and single-exposure analyses. The resulting estimates provide lead time for surveillance guided by forecasts and for region-specific targeting, supporting the use of seasonal climate predictability in TB control. From a One Health perspective, ENSO and local meteorological conditions represent upstream environmental signals that may shape the context in which infectious disease burden varies over time. By linking these environmental signals with TB surveillance data, this study examines the environmental and human health interface of One Health and evaluates the potential value of climate information as a supplementary component of climate sensitive TB surveillance.

## 2 Materials and methods

### 2.1 Study area and data sources

Data were assembled spanning January 2005 to December 2020, covering all 31 provincial-level administrative divisions (provinces, autonomous regions, and municipalities) in mainland China. This integrated dataset combined reported TB cases with key meteorological and environmental parameters, enabling a thorough examination of whether the ENSO and related climatic drivers influence TB epidemiological patterns in the region.

TB case data were sourced from the Tuberculosis Information Management System of the Chinese Center for Disease Control and Prevention (CDC) and its Public Health Science Data Center [24]. Launched in January 2005 by the former Ministry of Health of the People's Republic of China (now the National Health Commission), this system operates as the national mandatory platform for infectious disease surveillance. It aggregates and validates pulmonary TB notifications via the nationwide infectious disease reporting network. Organized at the provincial scale, these records encompass monthly and age-stratified case counts, delivering extensive spatial coverage, continuous temporal series, and standardized methodologies [25]. The analysis was conducted across 31 provincial level administrative divisions in mainland China, which represent the finest spatial unit at which standardized monthly TB surveillance data were consistently available nationwide throughout the study period. These features offer a dependable platform for evaluating TB trends and intervention efficacy. Over a long observation period, temporal variation in diagnostic practices, reporting completeness, and case detection capacity may affect the comparability of notification based incidence across provinces and years, and this should be considered when interpreting effect magnitude. Given that the present study uses provincially aggregated surveillance data, it is not possible to classify notified cases according to individual level disease history, such as primary progression, reinfection, or reactivation of latent TB, nor to distinguish cases detected through screening from those identified via routine clinical presentation.

The ENSO index was quantified using the Oceanic Niño Index (ONI), supplied by the United States National Oceanic and Atmospheric Administration (NOAA) (https://www.cpc.ncep.noaa.gov). El Niño events were defined as periods with ONI values at or above +0.5 °C, while La Niña events were defined as periods with ONI values at or below −0.5 °C, each persisting for at least five consecutive overlapping three month periods, in accordance with NOAA operational criteria. Calculated as the three-month running mean of sea surface temperature (SST) anomalies in the equatorial Pacific Niño 3.4 region (5°N-5°S, 120°-170°W) relative to a 30-year climatological reference, this index serves as a cornerstone for monitoring ENSO onset, development, and strength [26], [27]. These thresholds were adopted to ensure consistency with established operational definitions and to facilitate comparability with previous ENSO related epidemiological and climatological studies. It is routinely applied to define and categorize El Niño and La Niña events, including their intensity categories. ENSO intensity was further classified as weak, moderate, strong, and very strong according to increasing ONI magnitude, following standard ONI based classification conventions used in climate monitoring.

The meteorological variables (monthly mean temperature, relative humidity, and surface wind speed) were extracted from the ERA5-Land reanalysis dataset generated by the European Centre for Medium-Range Weather Forecasts (ECMWF) (https://www.ecmwf.int) [28]. Renowned for its elevated spatiotemporal resolution and internal consistency, this dataset aligns seamlessly with the scale of the TB records, supporting in-depth assessments of combined effects from ENSO and weather factors on TB occurrence.

To mitigate confounding from demographic and socioeconomic heterogeneity, adjustments were made for population density and per capita gross domestic product (PGDP) as covariates, with values obtained from the National Bureau of Statistics (https://data.stats.gov.cn). In addition, given air pollution's role in heightening susceptibility to respiratory infections, PM_2.5_ concentrations were included as an adjuster. Sourced from the ChinaHighAirPollutants (CHAP) dataset, this variable delivers a detailed portrayal of fine particulate matter pollution across mainland China [29], [30]. Merging these varied data streams effectively addressed disparities in population magnitude, economic progress, and air quality, thereby strengthening the robustness of parameter estimates and the credibility of causal interpretations.

The study region was segmented according to China's conventional seven geographical zones (North China, Northeast China, East China, Central China, South China, Southwest China, and Northwest China), utilizing provincial divisions as core analytical units [31], [32]. Incorporating topographic, climatic, and socioeconomic elements (Fig. 1), this classification provides a robust basis for regional subgroup investigations, revealing spatial variations in the effects of ENSO and meteorological influences on TB incidence.

**Fig. 1:**
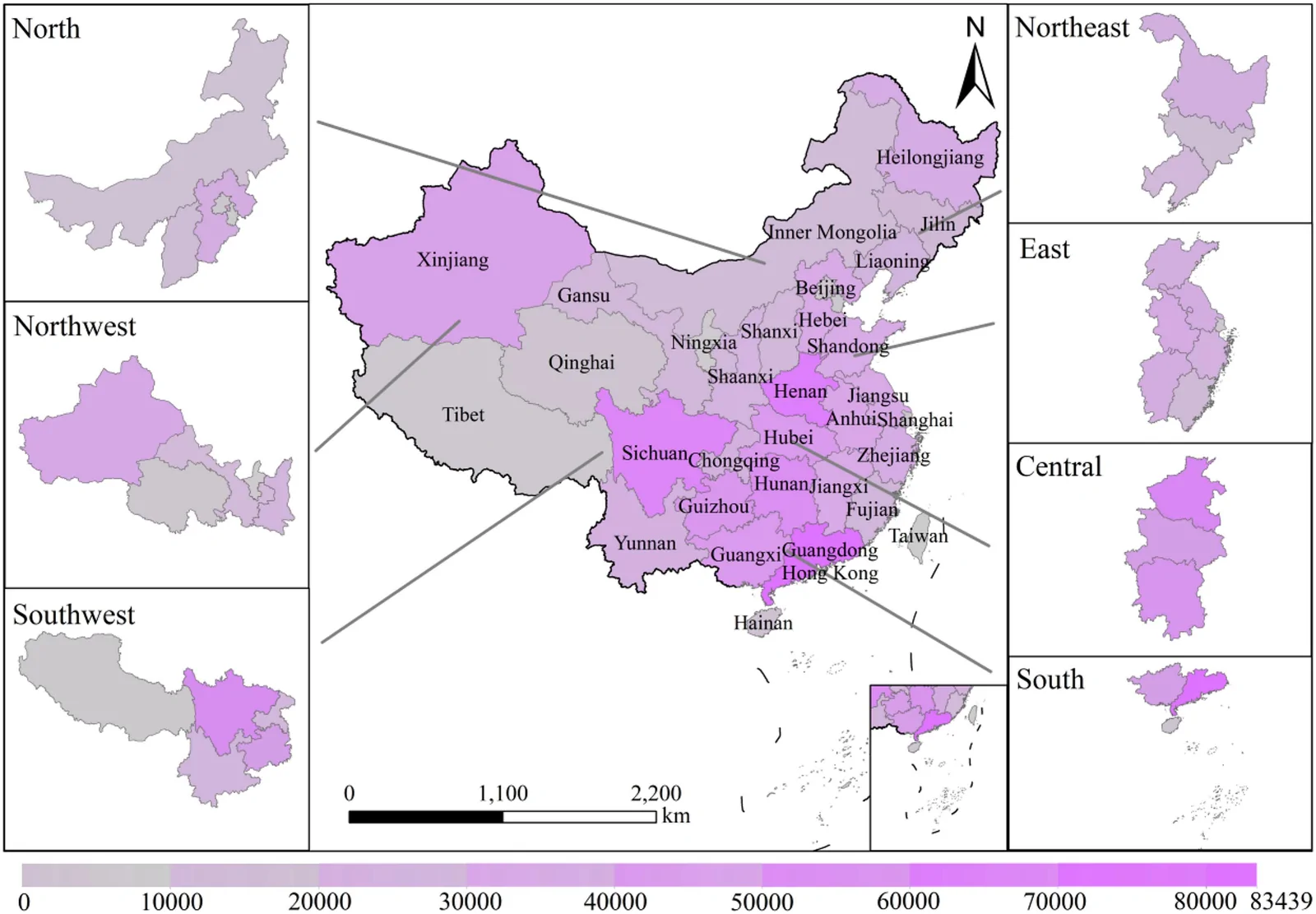
Spatial distribution of mean annual reported TB cases in mainland China. Fig. 1 displays the mean annual number of reported TB cases for each of the 31 provincial-level administrative divisions in mainland China, computed as the arithmetic mean of annual provincial case counts over 2005–2020. Insets delineate the seven conventional geographical regions (North, Northeast, East, Central, South, Southwest, and Northwest). Shading encodes provincial case-count ranges as defined in the color legend. Hong Kong, Macao, and Taiwan are shown for geographical reference only and were not included in the analytic dataset.

### 2.2 Definition and intensity classification of ENSO events

The ONI was adopted to define and classify ENSO events and their intensities [33]. Calculated as the three-month running mean of SST anomalies in the Niño 3.4 region (5°N-5°S, 120°-170°W) of the equatorial Pacific, the ONI serves as a principal indicator of ENSO-driven tropical climate variability [34]. Following the conventional NOAA ONI criteria, an El Niño event was defined as ONI values ≥ + 0.5 °C persisting for five consecutive three-month periods, while a La Niña event was defined as ONI values ≤ − 0.5 °C over the same duration; neutral conditions were defined as ONI values between −0.5 °C and + 0.5 °C [33]. Events are further categorized by intensity: weak (0.5–0.9 °C), moderate (1.0–1.4 °C), strong (1.5–1.9 °C), or very strong (≥2.0 °C) [35]. These categories require at least three consecutive three-month periods to meet the respective thresholds.

Given that ENSO related teleconnection patterns and associated atmospheric responses are most prominently characterized during the Northern Hemisphere winter (December–February, DJF), we summarized ENSO conditions using the DJF averaged ONI to represent boreal winter ENSO conditions in the main analyses [36], [37], [38]. To account for lagged effects and interannual variability in climate-TB relationships, a sensitivity analysis incorporated the annual mean ONI calculated from July of the previous year to June of the current year. This July–June window reflects the fact that ENSO conditions typically evolve over consecutive overlapping three month periods and frequently span two calendar years, thereby capturing interannual variability across seasons beyond a single winter summary. This classification framework facilitates the analysis of spatiotemporal impacts of ENSO driven climate patterns on TB incidence.

### 2.3 Statistical analysis

Distributed lag non-linear models (DLNMs) are widely employed in environmental epidemiology and public health to investigate complex, non-linear, and lagged associations between environmental exposures and health outcomes [9]. These models effectively capture the delayed effects of climatic variables, such as temperature or humidity, on disease dynamics by incorporating spline functions and lag structures. DLNMs enable joint modeling of exposure-response and lag-response relationships, accommodating temporal variations in effect magnitude, which is critical for count data exhibiting overdispersion, such as TB case notifications.

Prior research has indicated that extreme meteorological conditions and the ENSO can adversely affect population health, potentially modulating infectious disease transmission through environmental changes that influence pathogen survival and host susceptibility [11], [12], [13], [39]. In this study, a quasi-Poisson regression framework integrated with a DLNM was applied to examine the relationships between meteorological factors, the ENSO index, and TB incidence, supporting precise estimation of both immediate and delayed effects. The primary model is specified as follows:

(1) $$\mathrm{Log}\left[E\left(Y_{\mathrm{it}}\right)\right]=a+\mathrm{cb}\left(\mathrm{valu}e_{\mathrm{it}}\right)+\mathrm{ns}\left(\mathrm{RH},\mathrm{df}\right)+\mathrm{ns}\left(PM_{2.5},\mathrm{df}\right)+\mathrm{ns}\left(wi\mathrm{nd},\mathrm{df}\right)+\mathrm{ns}\left(\mathrm{Tim}e_{i},\mathrm{df}*\mathrm{year}\right)+{\mathrm{PD}}_{\mathrm{it}}+{\mathrm{PGDP}}_{\mathrm{it}}+{\mathrm{Region}}_{i}$$

In the model, *Y*_*it*_ represents the number of cases in province *i* on month *t*; *a* is the intercept; *cb* represents the cross-basis for mean temperature (Tmean), relative humidity (RH), and the ENSO, the exposure-response relationship was constructed using a B-spline function and the lag-response structure using a polynomial function. ENSO was modeled as an eight-level categorical exposure, including neutral conditions and seven observed El Niño and La Niña intensity categories, with neutral conditions serving as the reference. An “integer” function was specified for the exposure-response dimension of the cross-basis. *ns* represents the natural cubic spline function with 3 degrees of freedom (df). When analyzing the ENSO index, PM_2.5_ (df = 2), RH (df = 2), and wind speed (df = 4) were included as confounding factors in the model; Long-term and seasonal time trends were controlled for using a natural cubic spline with 3 df per year. PD represented population density; PGDP denoted regional GDP per capita, serving as an indicator of socioeconomic status; region_*i*_ represents a categorical variable for province. All model parameters were determined after considering the Akaike Information Criterion (AIC). Additionally, subgroup analyses were performed stratified by geographic region (North, Northeast, East, Central, South, Southwest, and Northwest) and age group cases (0–14 years,15–49 years, 50–69 years, and ≥ 70 years). Region-stratified exposure-response functions were derived within the same modeling framework using identical model specifications, and were intended to characterize geographic effect modification rather than to generate an aggregated summary estimate across regions.

### 2.4 Sensitivity analysis

To verify the stability of estimated associations between the ENSO, meteorological factors, and TB incidence, sensitivity analyses were conducted by varying key model specifications. Degrees of freedom for meteorological factors and long-term time trends (2–4 df each) were tested to assess model fit sensitivity. Control for confounders was implemented through single-factor and multi-factor models, including socioeconomic and environmental factors such as population density, PGDP, and PM_2.5_ concentrations, to evaluate their impact on primary results. Given that TB notifications in 2020 may have been affected by pandemic-related changes in healthcare access, we conducted a sensitivity analysis excluding this period. Detailed specifications are provided in the supplementary material (Table S2).

Statistical analyses were performed using R software (version 4.3.3), with the “dlnm” and “splines” packages applied to fit distributed lag non-linear models. We adopted a two-sided *P*-value threshold of <0.05 to determine statistical significance.

## 3 Results

### 3.1 Descriptive analysis

Over 2005–2020, monthly provincial TB case counts averaged 2569, with substantial variation across provinces (Table 1). All subsequent analyses are based on provincial TB notification counts derived from routine surveillance reporting. Age-stratified analysis showed that individuals aged 15–49 years consistently bore the highest burden, followed by those aged 50–69 years, whereas children (0–14 years) and older adults (≥70 years) accounted for comparatively fewer cases.

**Table 1:** Descriptive statistics.

	Mean	Standard Deviation	Minimum	Maximum	Median
All Cases	2569.28	1875.28	26.00	17,549.00	2309.50
Age group cases					
0–14 years	29.80	40.41	0.00	382.00	17.00
15–49 years	1340.98	1020.30	19.00	7585.00	1185.50
50–69 years	825.95	611.53	5.00	6458.00	716.00
≥70 years	372.52	297.81	1.00	3122.00	295.50
ONI	−0.06	0.84	−1.60	2.60	−0.20
PM_2.5_	43.54	25.67	5.70	192.20	35.80
Tmean	11.92	11.27	−23.04	30.18	13.74
RH	63.41	15.73	21.37	90.12	67.23
Wind	0.91	0.56	0.00	3.85	0.81
PD	2716.60	1259.38	188.52	6307.38	2496.39
PGDP	41,860.22	27,344.42	5218.00	164,158.00	35,991.00

Table 1 presents the summary statistics (mean, standard deviation, minimum, maximum, median) for all variables used in the study. The data are monthly aggregates at the provincial-administrative level in mainland China from 2005 to 2020.

Case refers to the monthly count of newly reported TB cases.

Age-stratified cases are disaggregated into four groups (0–14, 15–49, 50–69, and ≥70 years).

ONI (Oceanic Niño Index) is based on sea surface temperature anomalies in the Niño 3.4 region. Warm (cold) phases are defined by thresholds of ≥ + 0.5°C (≤ − 0.5°C) sustained for at least five consecutive overlapping 3-month periods.

PM_2.5_ represents the monthly average concentration of fine particulate matter (μg/m^3^).

Tmean denotes mean air temperature at 2 m above ground (°C).

RH indicates relative humidity (%).

Wind refers to ground-level wind speed (m/s).

PD is population density (persons/km^2^).

PGDP represents per capita gross domestic product (CNY/person).

The correlation structure among ENSO and key meteorological variables is summarized in Fig. S2.

To facilitate regional comparability, the spatiotemporal distribution of provincial TB incidence rates in selected years is presented in Fig. S3.

Spatial heterogeneity in TB case counts was evident, with higher mean annual values observed in central, southern, and certain southwestern provinces (Fig. 1). Differences among the seven geographical regions reflected substantial spatial imbalance in TB case distribution.

Temporal trends showed a general decline in TB cases, which is consistent with the long-term investment and enhanced control measures implemented by the Chinese government in TB prevention and treatment (Fig. 2). Age-stratified trends paralleled this overall decrease.

**Fig. 2:**
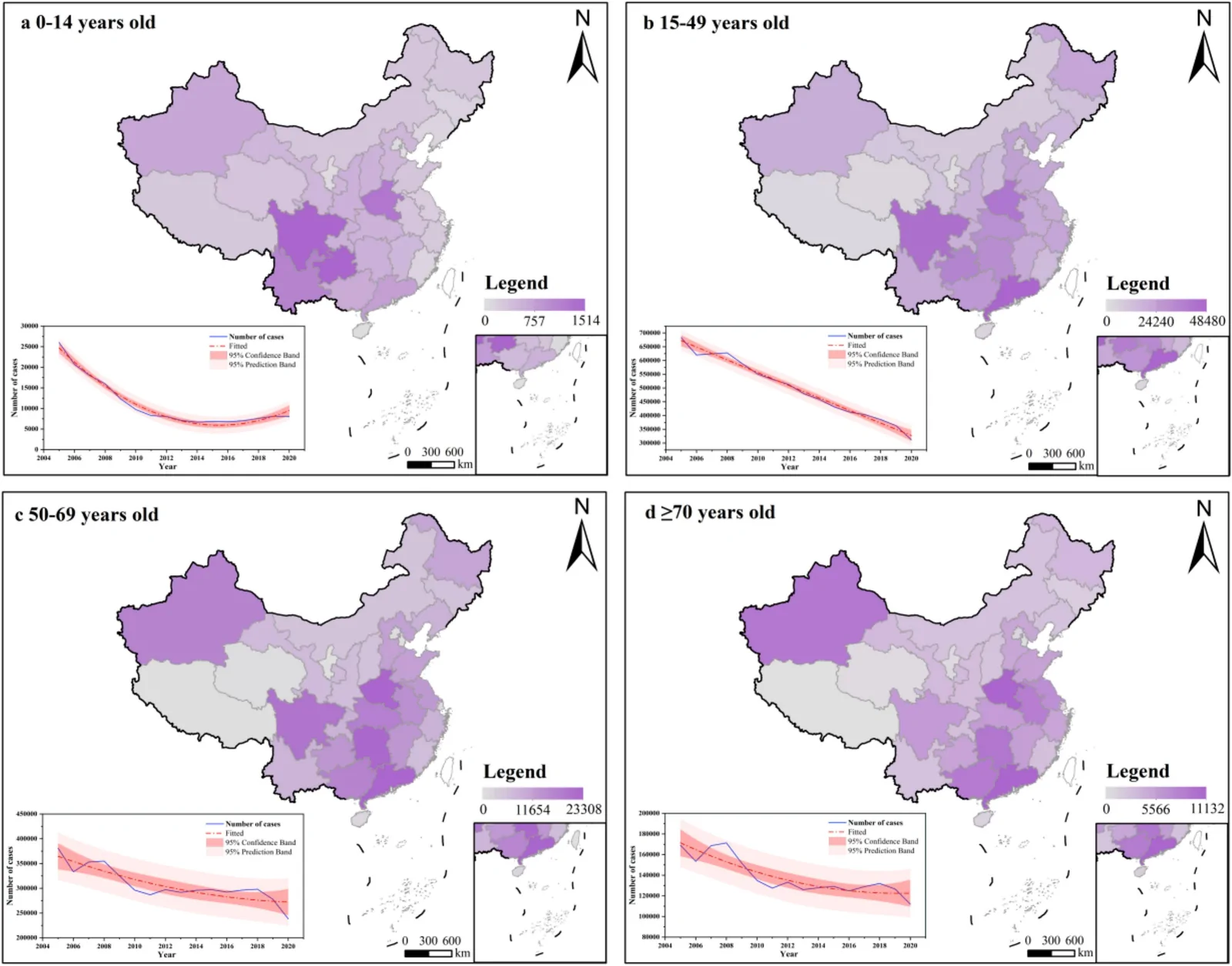
Age-stratified number of reported TB cases across mainland China, 2005–2020. Fig. 2, Panels (a-d), Spatiotemporal distribution of TB cases based on national surveillance data, presented by age group. Panel a illustrates the 0–14 years age group, panel b 15–49 years, panel c 50–69 years, and panel d ≥ 70 years. In each panel, the solid blue line represents the observed monthly number of reported cases, while the shaded red area indicates the uncertainty band around the smoothed temporal trend.

ENSO phases, classified using ONI thresholds, indicated La Niña conditions in 32.81% of study months and El Niño conditions in 25.52%. Weak El Niño and weak La Niña conditions were the most frequent intensity categories, accounting for 18.23% and 23.44% of all study months, respectively, whereas strong and very strong conditions were less common (e.g., very strong El Niño: 3.13%; strong La Niña: 3.13%; Table S1).

### 3.2 Association between TB incidence and ENSO and meteorological factors

Distributed lag nonlinear models identified significant exposure-response relationships between meteorological variables, ONI, and TB incidence (Fig. 3). Temperature exhibited a U-shaped association with TB risk, with both low and high temperatures associated with increased incidence. The adverse effects were particularly pronounced at higher temperatures. For relative humidity, a significant risk increase was observed within the range of 67% to 87%, peaking at 81% (RR = 1.101; 95% CI: 1.063–1.140). ENSO effects showed a delayed response, with the strongest association observed at lag 10 months. Compared with neutral conditions, the estimated RR at lag 9 months was 1.048 for strong El Niño (95% CI: 1.007 to 1.091) and 1.058 for strong La Niña (95% CI: 1.032 to 1.085).

**Fig. 3:**
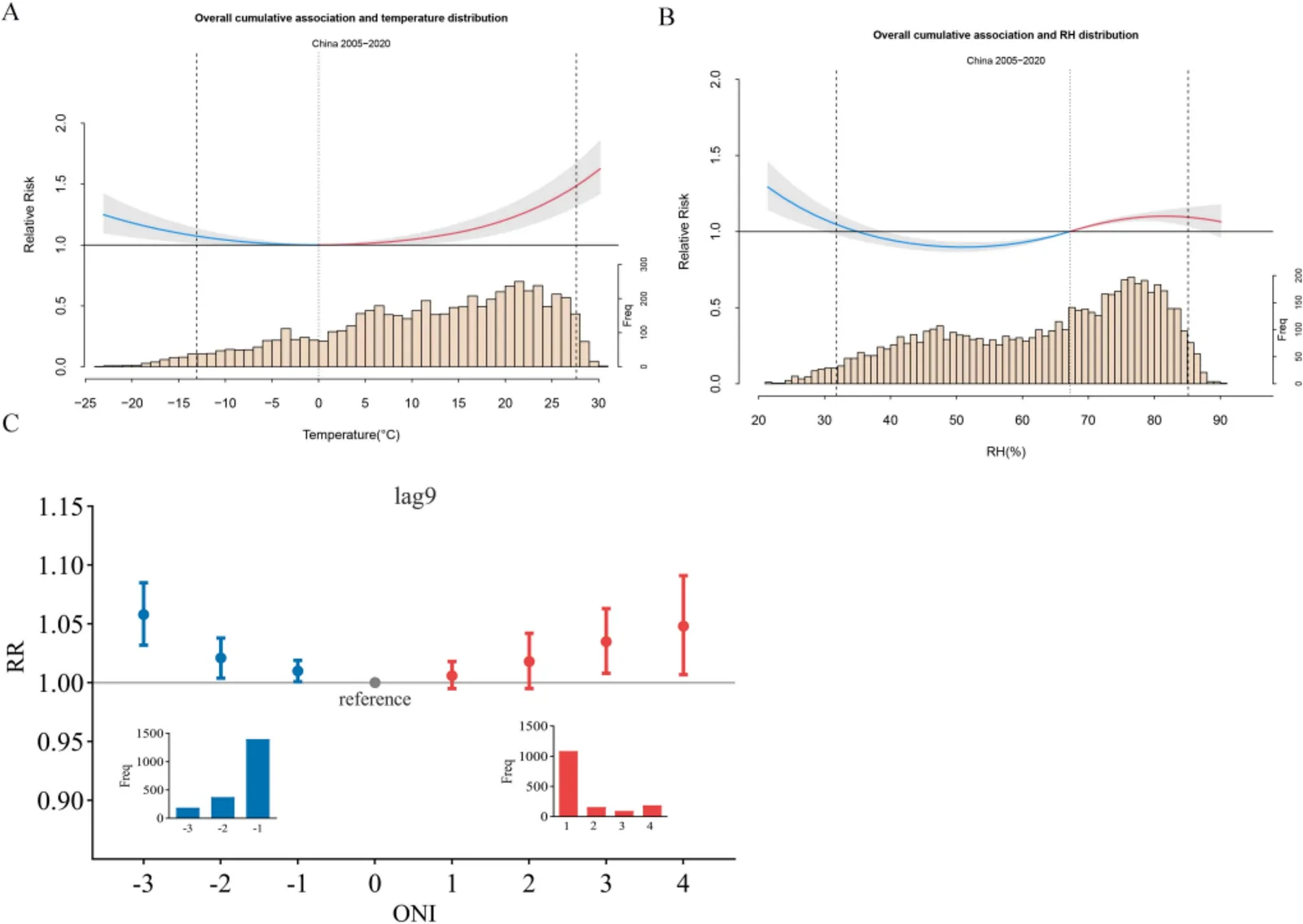
Cumulative exposure-response relationships between meteorological factors, ENSO, and TB incidence. Fig. 3 presents the overall cumulative exposure-response associations of temperature, RH, and the ONI with TB incidence, estimated using DLNMs. Panel A shows temperature; Panel B shows RH; Panel C shows lag specific RRs for ONI categories at lag 9 months, with neutral conditions as the reference. Solid lines denote estimated relative risks, the gray dashed line marks the 50th percentile as the reference level, and short black lines indicate the 2.5th and 97.5th percentiles for each exposure. Histograms show the exposure distributions. Temperature displays a U-shaped association with higher risk at both low and high values. For relative humidity, risk increases between 67% and 87%, peaking at 81% (RR: 1.101; 95% CI: 1.063–1.140). At lag 9 months, strong El Niño and strong La Niña categories were associated with higher TB risk than neutral conditions.

### 3.3 Lag effects of meteorological factors and ENSO

DLNMs revealed significant delayed effects of meteorological variables and ENSO on TB incidence over lag periods of 0–12 months (Fig. 4). Extreme heat showed the most pronounced association with TB risk at lag 0, with elevated risks persisting for up to 6 months. Extreme cold was also associated with increased TB risk for approximately 6 months.

**Fig. 4:**
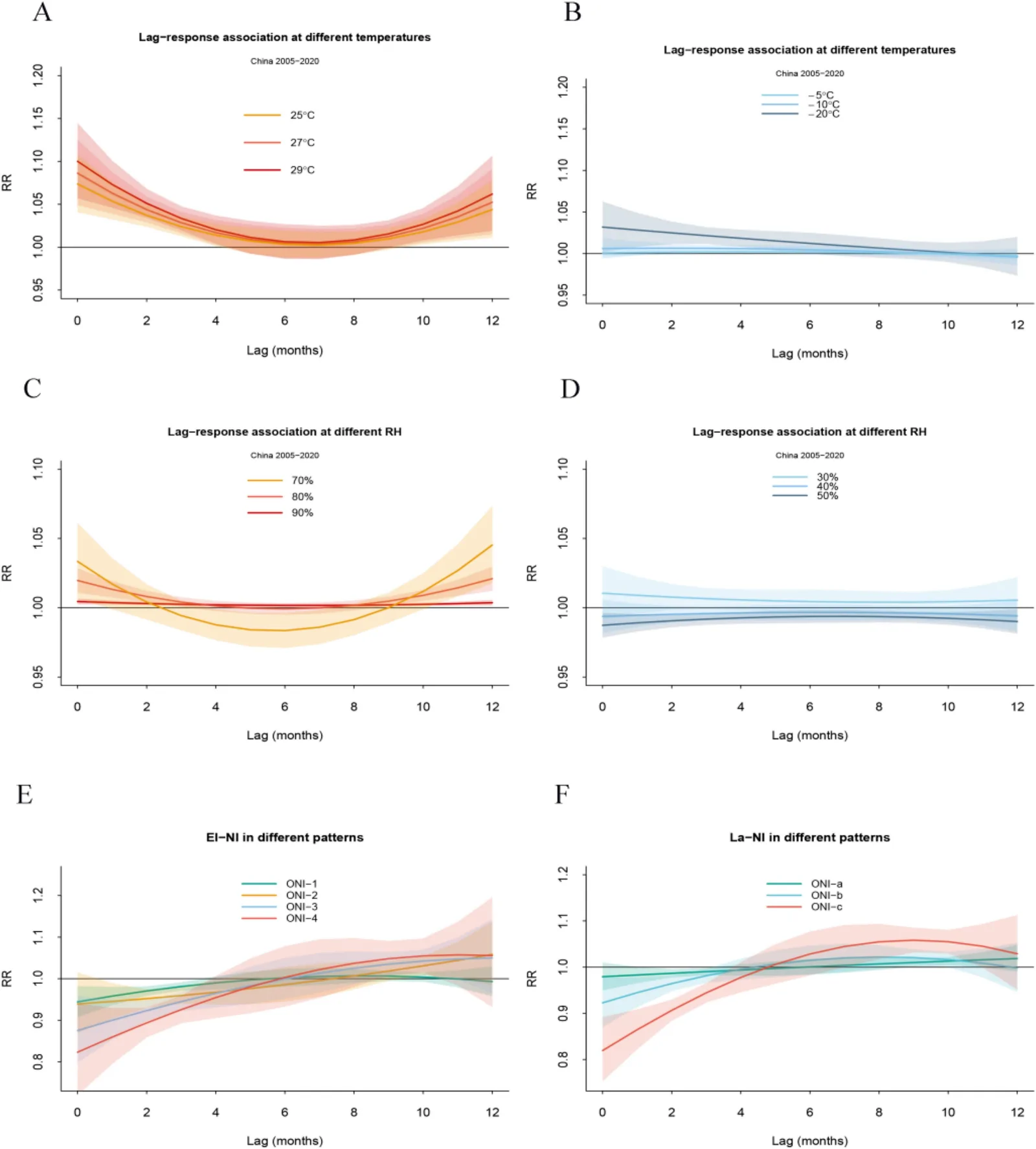
Lagged effects of meteorological factors and ENSO on TB incidence. Fig. 4 Lagged RR of TB incidence associated with temperature, RH, and the ONI over lags 0 to 12 months, derived from distributed lag nonlinear models. Extreme heat showed the strongest RR at lag 0, with elevated RRs persisting for up to 6 months. Increased RRs under cold conditions persisted for approximately 6 months. Moderate RH from 67% to 87% with a peak at 81% was associated with increased risk beginning at lag 0 and lasting nearly 2 months. ENSO related associations began to emerge at lag 9 months, reached the strongest association at lag 10 months, and persisted for approximately 3 months. Both strong El Niño and strong La Niña phases were associated with higher TB risk compared with neutral conditions. Panels show: (A) warm temperature conditions; (B) cold temperature conditions; (C) high RH conditions; (D) low RH conditions; (E) El Niño phases; (F) La Niña phases. ONI category labels are defined as follows: weak (ONI-1/+0.5–0.9 °C; ONI-a/−0.5 to −0.9 °C), moderate (ONI-2/+1.0–1.4 °C; ONI-b/−1.0 to −1.4 °C), strong (ONI-3/+1.5–1.9 °C; ONI-c/≤ − 1.5 °C), and very strong (ONI-4/≥ + 2.0 °C).

Moderate to high relative humidity within the elevated-risk range of 67% to 87%, peaking near 81%, was associated with an immediate increase in TB risk at lag 0, lasting approximately 2 months. ENSO related associations emerged around lag 9 months, reached the strongest association at lag 10 months, and persisted for approximately 3 months. Both strong El Niño and strong La Niña events were associated with higher TB incidence compared with neutral conditions.

### 3.4 Subgroup analysis

Subgroup analyses revealed variations in exposure-response relationships between meteorological factors and TB incidence across geographic regions and age strata (Fig. 5). The regional curves suggest interval-specific associations and region-specific exposure thresholds, consistent with effect modification across heterogeneous climatic contexts. Analyses stratified by China's seven geographical regions demonstrated U- or J-shaped associations for both temperature and RH. Higher estimated RRs at the upper temperature range were mainly observed in North China and Southwest China, whereas higher estimated RRs at the lower temperature range were more apparent in Northeast China and Northwest China. For RH, higher estimated RRs at low humidity were most evident in Southwest China and Central China. At high RH, the upward pattern was strongest in Central China and was also observed in Southwest China, with a less pronounced upward trend in South China at the upper end of the RH range.

**Fig. 5:**
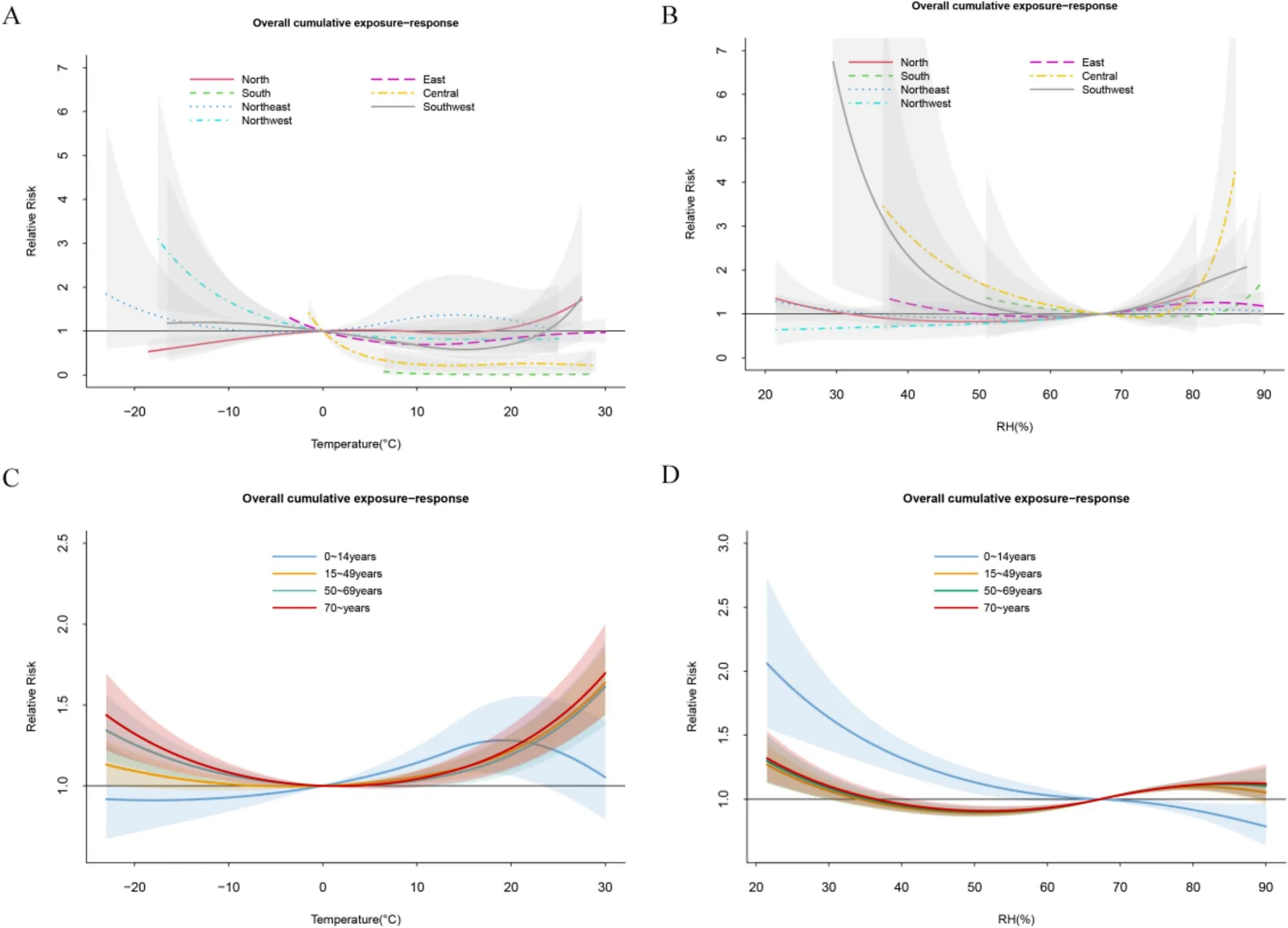
Cumulative exposure-response curves of meteorological factors and TB incidence, by geographic region (a) and age group (b). Fig. 5 depicts stratified exposure response relationships between temperature, RH, and TB incidence across subgroups. Panels A and B present region stratified results across seven geographical regions. For temperature, higher estimated RRs at the upper temperature range were mainly observed in North China and Southwest China, whereas higher estimated RRs at the lower temperature range were more apparent in Northeast China and Northwest China. For RH, higher estimated RRs at low humidity were mainly observed in Southwest China and Central China. At the upper RH range, the upward pattern was most pronounced in Central China, was also observed in Southwest China, and appeared less pronounced in South China. Panels C and D display age stratified associations. Temperature related increases at both low and high extremes were more evident among adult and older age groups than among children aged 0 to 14 years. For RH, children aged 0 to 14 years showed the most pronounced increase at low humidity, whereas modest increases at higher RH were mainly observed among adult and older age groups.

Age stratified analyses showed heterogeneous exposure response patterns across age groups. Temperature related increases at both low and high values were more evident among adult and older age groups than among children aged 0 to 14 years. For RH, children aged 0 to 14 years showed the most pronounced increase at low humidity, whereas modest increases at higher RH were mainly observed among adult and older age groups. Adults aged 15 to 49 years accounted for the largest number of notifications but did not necessarily show the highest exposure response estimates.

### 3.5 Sensitivity analysis

Sensitivity analyses were conducted to evaluate the robustness of the observed associations between ENSO, meteorological variables, and TB incidence. We varied the degrees of freedom (ranging from 2 to 4) for the natural spline functions applied to meteorological factors and long-term temporal trends to assess potential influences of smoothing specifications. Additionally, the results remained robust after excluding data from the 2020 pandemic period. Models were further adjusted for key potential confounders, including PM_2.5_, population density, PGDP, and an interaction term between province and the continuous time variable, to evaluate their impact on the effect estimates of primary exposures.

Sensitivity analysis indicated that the RRs calculated via different degrees of freedom for time trends (2–4 df), meteorological factors (2–4 df), and alternative controls for air pollutants in the models were similar. This indicated that this model was reliable and stable (Table S2). The exposure-lag-response relationships for these variables did not exhibit substantial changes, indicating that the primary findings are robust to variations in model parameterization and adjustment for socioeconomic and environmental covariates.

## 4 Discussion

### 4.1 Interpretation of findings

This study identified significant nonlinear and temporally delayed associations of ENSO and local meteorological factors with TB notification incidence across China. The U-shaped association between temperature and TB risk indicates that both cold and heat extremes elevate transmission potential. Cold conditions may prolong the viability of aerosolized *Mycobacterium tuberculosis* (MTB), while extreme heat may promote indoor crowding and compromise host immune function, thereby increasing exposure risk and susceptibility.

The higher TB notification incidence observed at relative humidity levels of 67% to 87%, peaking at 81%, may be consistent with humidity-related changes in respiratory droplet behavior and pathogen persistence. Such conditions may delay droplet evaporation without substantial dilution, potentially extending the infectious period. These findings align with established biological mechanisms underlying humidity-dependent survival of airborne pathogens [40], [41], [42].

A key finding is the delayed influence of ENSO on TB incidence, with the strongest effects observed at a 10-month lag and sustained for approximately 3 months. This lag structure is biologically and epidemiologically plausible, as ENSO-related climatic anomalies may affect TB risk through delayed pathways involving food insecurity and undernutrition, subsequent changes in host susceptibility, and downstream effects on transmission dynamics and case detection. This protracted pattern may reflect delayed pathways linking ENSO-related climatic anomalies with TB notification burden, including food insecurity, undernutrition, and health system disruptions during and after climate anomalies [12], [43], [44]. Age-stratified analyses indicated heterogeneous exposure-response patterns. Children aged 0 to 14 years showed a more pronounced increase in TB risk at low relative humidity, whereas temperature-related increases were more evident among adult and older age groups. Previous studies suggest that nutritional status and age-related host factors may contribute to childhood TB risk [45], [46]. However, these mechanisms were not directly evaluated in the present study.

This study extends the existing literature in several important respects. While Kovats et al. (2003) highlighted the role of ENSO in amplifying infectious disease outbreaks, their focus was primarily on vector-borne and diarrheal diseases, which typically exhibit shorter lag times [13]. In contrast, our findings reveal that TB, a chronic airborne disease, exhibits a more delayed response pattern, underscoring the importance of pathogen-specific mechanisms. Anttila-Hughes et al. (2021) reported an association between ENSO and child undernutrition [12]; our findings extend this literature by identifying a delayed association between ENSO and TB notification incidence. Similarly, while Xiao et al. (2022) identified meteorological mediation of ENSO effects on influenza, and Tian et al. (2025) reported associations between increasing ENSO amplitude and teleconnections and elevated global dengue risk [11], [47], our study identified a broader range of nonlinear associations and longer lag structures, which may reflect differences in disease natural history, transmission dynamics, and model specification. Beyond disease-specific outcomes, recent high-impact evidence has further demonstrated that ENSO variability can exert enduring and cumulative effects on population health, including long-term reductions in life expectancy under both historical and projected climate conditions, underscoring the broader and persistent health relevance of ENSO [48].

Notably, our use of DLNMs and nationwide data allowed detection of effect thresholds and delayed patterns that linear models or subnational studies may overlook [9], [49]. Furthermore, the symmetric increase in TB risk during both strong El Niño and strong La Niña events challenges the conventional focus on warm-phase events and suggests that both extremes of the ENSO spectrum can disrupt socioecological systems in ways that favor TB transmission [50], [51], [52].

### 4.2 Theoretical and practical implications

Theoretical implications of this research include the refinement of climate-health frameworks to account for nonlinear and lagged responses in bacterial respiratory infections. The demonstration that both phases of ENSO can increase TB risk supports a more integrated approach to studying climate teleconnections and infectious disease outcomes. These findings add an environmental surveillance dimension to One Health oriented TB control. ENSO and local meteorological variables are upstream climate signals that may provide lead time for strengthening TB surveillance before changes in notification burden become apparent.

From a public health perspective, the observed lagged associations suggest that seasonal climate information may have potential value as a supplementary input to TB surveillance and preparedness. The predictability of ENSO several months in advance provides an opportunity to evaluate whether ONI and related climate information can improve the timing of surveillance activities [53], [54]. These findings therefore support further evaluation of seasonal climate information, including ONI, as a supplementary component of TB surveillance and preparedness. Potential applications could include the evaluation of climate informed surveillance windows, enhanced diagnostic preparedness, and locally adapted public health responses. Such applications would require prospective validation of forecast performance, operational thresholds, and the added value of climate information beyond existing surveillance indicators. Implementation would also need to account for regional variation in forecast skill, workforce and laboratory capacity, and local decision making processes. In areas showing stronger climate associated variation in TB notification burden, preparedness measures could be considered alongside locally relevant surveillance indicators [55], [56]. Operational development of such an approach would require closer coordination between meteorological and public health surveillance systems, with future integration of relevant animal health and environmental data where available.

The age-stratified findings also highlight the importance of maintaining attention to pediatric TB surveillance and services, particularly given the more pronounced association with low relative humidity observed among children aged 0 to 14 years [57], [58]. Nutritional support remains relevant to TB prevention because undernutrition is an established risk factor for TB [22], [59], [60]. However, nutritional pathways were not directly evaluated in the present study and should not be inferred from the observed climate-related associations. More broadly, coordination among meteorological agencies, public health authorities, and relevant health services may facilitate the future evaluation of climate-informed approaches to TB surveillance [61].

### 4.3 Limitations

Several limitations must be considered. First, the ecological design of the study precludes causal inference at the individual level, and we were unable to adjust for certain confounders such as HIV prevalence, indoor air quality, or individual-level socioeconomic status [62]. TB notifications in 2020 may have been affected by pandemic-related changes in healthcare access, diagnostic activity, mobility, reporting, and surveillance. To assess the potential impact of this bias, we conducted a sensitivity analysis excluding all 2020 data. The results were consistent with the primary analysis, suggesting that the observed associations are not solely driven by pandemic-period data. Nevertheless, residual bias from pandemic-related disruptions cannot be entirely ruled out. Another important limitation concerns outcome granularity. TB comprises biologically and epidemiologically distinct pathways, including primary disease progression, reinfection, and reactivation of latent infection, and the relative contribution of these mechanisms may vary across settings. TB notification incidence was calculated as the number of monthly notified pulmonary TB cases divided by the corresponding provincial population and expressed per 100,000 population. Specifically, the analytical models were fitted using monthly provincial TB notification counts, with population size accounted for through a log population offset, and the term incidence is used to denote population level notification burden rather than individually ascertained risk. Such etiologic heterogeneity may attenuate or modify the estimated associations if different pathways respond differently to climatic exposures. Accordingly, the findings should be interpreted as reflecting associations with the overall provincial TB notification burden rather than with specific mechanisms of disease occurrence. Second, although province-level analysis offers broad coverage, it may mask subprovincial heterogeneity in climate, infrastructure, and TB burden [63]. Province level administrative units were selected as the most consistent spatial scale at which standardized TB surveillance data are available nationwide over long time periods. Third, while we focused on ENSO because of its prominent role in large-scale climate variability, other modes of oceanic and atmospheric variability may also influence infectious disease dynamics and were not examined in the present study [64]. Finally, underreporting and diagnostic delays in TB surveillance systems may have resulted in underestimation of true incidence, particularly in remote regions [65]. Such biases may reflect variability in access to health services and case detection capacity across regions, and are more likely to bias estimates toward underestimation rather than generate spurious associations. Animal health data, zoonotic TB information, and pathogen typing were not available in this national surveillance analysis, precluding direct assessment of animal reservoirs or zoonotic transmission pathways. Accordingly, the One Health relevance of the present study primarily concerns the environmental dimension of human TB surveillance. Future studies integrating human TB notifications with animal health, pathogen, and environmental data could provide a more comprehensive assessment of climate sensitive TB dynamics across the human, animal, and environmental interface.

### 4.4 Future research directions

We prioritize future research directions according to expected public health utility and feasibility of near term implementation. Future studies should aim to integrate individual-level data on nutrition, immune status, and behavior to better elucidate the mechanisms linking climate variability to TB outcomes [56]. Research using cohort or case-control designs would strengthen causal inference. Spatially granular analyses (at the county or city level) could help identify local risk hotspots and improve targeting of interventions. Future studies should leverage daily resolved meteorological data to derive cold spell related indices and evaluate their independent and joint associations with ENSO phase and intensity, thereby enhancing the practical value of climate informed surveillance planning. Incorporating additional climate indices and employing machine learning methods that account for climate lag effects may further improve predictive accuracy [66]. Finally, investigating the effects of climate variability on TB treatment outcomes and drug resistance patterns would offer valuable insights for building climate-resilient TB control programs.

## 5 Conclusion

Using 16 years of national surveillance data from China, this study characterized nonlinear and delayed associations of ENSO and meteorological factors with TB notification incidence using DLNMs. Temperature showed a U-shaped association, while relative humidity of 67% to 87% was associated with higher TB notification incidence, peaking at 81% (RR, 1.101; 95% CI, 1.063 to 1.140). ENSO-related associations peaked at lag 10 months and persisted for approximately 3 months, whereas temperature-related associations persisted for up to 6 months. Both strong El Niño and strong La Niña conditions were associated with higher TB notification incidence, while regional and age-stratified analyses indicated heterogeneous exposure-response patterns. These findings support further evaluation of climate forecasts as supplementary information for climate-sensitive TB surveillance. From a One Health perspective, the study highlights the potential value of incorporating environmental information into human infectious disease surveillance and underscores the importance of future integration with relevant animal health, pathogen, and environmental data.

## CRediT authorship contribution statement

**Xiaopeng Qin:** Writing – original draft, Data curation. **Yizhang Xia:** Writing – original draft, Supervision, Data curation. **Jiang Xue:** Resources, Methodology. **Xudong Zhou:** Data curation. **Wenjie Tao:** Validation, Methodology. **Yukang Wu:** Funding acquisition, Data curation, Conceptualization. **Hongchang Zhang:** Data curation. **Ying Chen:** Visualization. **Zhenyu Lin:** Supervision. **Lanwei Nong:** Data curation. **Kechang He:** Software. **Zhendong Qin:** Validation, Conceptualization. **Tiejun Zhou:** Methodology, Formal analysis. **Zhouhua Xie:** Writing – review & editing, Project administration. **Jie Ma:** Writing – review & editing, Project administration.

## *Consent for publication*

Not applicable.

## *Ethics approval and consent to participate*

This study was performed in line with the principles of the Declaration of Helsinki. Approval was granted by the Ethics Committee of the Fourth People's Hospital of Nanning (No. 2023-36-01).

## Ethics declaration

The authors have NOT obtained informed consent from participants or their legal representatives. This study used routinely collected, aggregated tuberculosis surveillance data and involved no direct contact with individual participants. The requirement for individual informed consent was waived by the Ethics Committee of the Fourth People's Hospital of Nanning (Approval No. 2023-36-01). The analysis was conducted using population-level surveillance data, and no personally identifiable information was included in the study.

This study was performed in compliance with relevant laws, regulatory frameworks and guidelines where the research took place. This study was approved by the Ethics Committee of the Fourth People's Hospital of Nanning. (Approval No. 2023-36-01)

## Funding

This study was supported by Innovation Project of Guangxi Graduate Education (Grant number: YCBZ2025134). Funding bodies had not participated in the design of the study, collection, interpretation, and analysis of the data, or in writing the manuscript.

## Declaration of competing interest

The authors report there are no competing interests to declare.

## Data Availability

The original contributions presented in the study are included in the article/Supplementary Material; further inquiries can be directed to the corresponding authors.
