# Supplementary material for: Associations between El Niño-Southern Oscillation (ENSO) and tuberculosis incidence in China: a nationwide study
Source: One Health. 2026 Sep 16;23:101587. doi: 10.1016/j.onehlt.2026.101587 (PMC13627933; doi:10.1016/j.onehlt.2026.101587)
Supplement: Supplementary file 1 [file mmc1.docx]

**Supporting information for**

**Associations between El Niño-Southern Oscillation (ENSO) and Tuberculosis Incidence in China: A Nationwide Study**

**Note**

Due to restrictions in data availability, the regions of Hong Kong, Macau, and Taiwan are excluded from the study area.

The maps in this study were generated using ArcGIS software (version 10.7, Esri, USA) with base map data sourced from the Administrative Divisions Information Platform of the Ministry of Civil Affairs of the People's Republic of China, under map approval number GS (2024) 0650.

**Outcome definition and data structure**

TB outcomes were defined using provincially aggregated notification data obtained from the Tuberculosis Information Management System of the Chinese Center for Disease Control and Prevention. The outcome represents provincial TB notification incidence derived from routine surveillance reporting. Because the data used in this study are aggregated at the provincial level, individual level disease histories are not available, and cases cannot be classified by disease occurrence mechanisms such as primary progression, reinfection, or reactivation of latent infection, nor by mode of detection.

**Supplementary Table S1~2**

Table S1: Distribution of ENSO Event Classifications Based on the ONI.

Table S2: Sensitivity analysis of the model (time trend freedom 2-4, meteorological factors trend freedom 2-4 and alternative controls).

**Figure S1~3**

Figure S1 The stratified exposure-response relationships between temperature and TB incidence across seven geographical regions.

Figure S2 Spearman correlation analysis of meteorological variables and ENSO. Figure S3 Spatiotemporal distribution of tuberculosis incidence rates across mainland China in selected years (2005, 2010, 2015, and 2020).

**Table S1. Distribution of ENSO Event Classifications Based on the ONI**

| ONI classification | Frequency | Percent |
| --- | --- | --- |
| Total | 192 | 100.00% |
| El Niño | 49 | 25.52% |
| Weak (ONI-1) | 35 | 18.23% |
| Moderate (ONI-2) | 5 | 2.60% |
| Strong (ONI-3) | 3 | 1.56% |
| Very Strong (ONI-4) | 6 | 3.13% |
| Neutral conditions | 80 | 41.67% |
| La Niña | 63 | 32.81% |
| Weak (ONI-a) | 45 | 23.44% |
| Moderate (ONI-b) | 12 | 6.25% |
| Strong (ONI-c) | 6 | 3.13% |

Note: El Niño events were defined by ONI ≥+0.5°C and La Niña events by ONI≤–0.5°C, each persisting for at least five consecutive overlapping three-month periods. Intensities were classified as weak (ONI-1/+0.5-0.9°C; ONI-a/–0.5 to –0.9°C), moderate (ONI-2/+1.0-1.4°C; ONI-b/–1.0 to –1.4°C), strong (ONI-3/+1.5-1.9°C; ONI-c/≤–1.5°C), and very strong (ONI-4/≥+2.0°C). Neutral conditions (−0.5°C < ONI < +0.5°C). Frequencies denote the number of months classified into each ENSO category during the study period from 2005 to 2020; percentages represent the corresponding proportion of all months.

**Table S2. Sensitivity analysis of the model (time trend freedom 2-4, meteorological factors trend freedom 2-4 and alternative controls)**

| Model ^a^ | Value | df | Overall CRR and 95%CI | |
| --- | --- | --- | --- | --- |
|  |  |  | P_2.5% effect (ENSO,4) | P_97.5% effect  (ENSO, -3) |
| Model1 | Time | 3 | 1.218(1.082,1.370) | 1.547(1.362,1.756) |
|  |  | 4 | 1.233(1.101,1.387) | 1.163(1.025,1.319) |
|  |  | 5 | 1.257(1.116,1.417) | 1.162(1.004,1.344) |
|  | RH | 2 | 1.218(1.082,1.370) | 1.547(1.362,1.756) |
|  |  | 3 | 1.217(1.081,1.370) | 1.547(1.362,1.756) |
|  |  | 4 | 1.217(1.081,1.370) | 1.554(1.367,1.766) |
|  | PM_2.5_ | 2 | 1.218(1.082,1.370) | 1.547(1.362,1.756) |
|  |  | 3 | 1.241(1.102,1.398) | 1.518(1.336,1.725) |
|  |  | 4 | 1.247(1.107,1.404) | 1.509(1.328,1.715) |
|  | wind | 2 | 1.219(1.083,1.371) | 1.547(1.363,1.756) |
|  |  | 3 | 1.217(1.082,1.370) | 1.548(1.363,1.757) |
|  |  | 4 | 1.218(1.082,1.370) | 1.547(1.362,1.756) |
|  | Without PM_2.5_ | / | 1.330(1.183,1.496) | 1.452(1.280,1.647) |
|  | Without PD | / | 1.218(1.081,1.372) | 1.543(1.358,1.754) |
|  | Without PGDP | / | 1.231(1.094,1.385) | 1.542(1.358,1.750) |
|  | Without 2020 year | / | 1.230(1.092,1.385) | 1.649(1.451,1.875) |
|  | factor(region):time^c^ | / | 1.065(0.960,1.182) | 1.064(1.028,1.101) |
| Model2-ENSO ^b^ | | | | |
| Model2 | Time | 2 | 1.055(1.015,1.097) | 1.058(1.032,1.085) |
|  |  | 3 | 1.035(0.997,1.073) | 1.046(1.021,1.070) |
|  |  | 4 | 1.037(0.999,1.075) | 1.043(1.019,1.067) |
|  | RH | 2 | 1.055(1.015,1.097) | 1.058(1.032,1.085) |
|  |  | 3 | 1.055(1.015,1.096) | 1.059(1.033,1.086) |
|  |  | 4 | 1.054(1.014,1.095) | 1.059(1.032,1.085) |
|  | PM_2.5_ | 2 | 1.055(1.015,1.097) | 1.058(1.032,1.085) |
|  |  | 3 | 1.054(1.014,1.095) | 1.059(1.032,1.086) |
|  |  | 4 | 1.055(1.014,1.096) | 1.060(1.033,1.086) |
|  | Without PM_2.5_ | / | 1.055(1.014,1.097) | 1.054(1.028,1.081) |
|  | Without PD | / | 1.054(1.014,1.096) | 1.059(1.032,1.086) |
|  | Without PGDP | / | 1.055(1.015,1.097) | 1.060(1.033,1.087) |
|  | Without 2020 year | / | 1.029(0.993,1.066) | 1.032(1.008,1.057) |
|  | factor(region):time^c^ | / | 1.057(1.024,1.090) | 1.045(1.023,1.067) |

Note: Model ^a^ (Model1, cb.tmean; Model2, cb.ENSO); CRR: cumulative relative risk; CI: confidence interval. ^b^ denotes the specific lag month within that model where the effect estimate reached its maximum and was statistically significant (e.g., lag 10 for ENSO-4 and lag 9 for ENSO-(-3)).^c^ an interaction term between province and the continuous time variable).

**Figure S1.**

Figure S1 depicts the stratified exposure-response relationships between temperature and TB incidence across seven geographical regions.

**Figure S2.**

Figure S2 Spearman correlation analysis of meteorological variables and ENSO.

**Figure S3.**

Figure S3 Spatiotemporal distribution of tuberculosis incidence rates across mainland China in selected years (2005, 2010, 2015, and 2020).
